# Supplementary material for: COZOID: contact zone identifier for visual analysis of protein-protein interactions
Source: BMC Bioinformatics. 2018 Apr 6;19:125. doi: 10.1186/s12859-018-2113-6 (PMC5889581; doi:10.1186/s12859-018-2113-6)
Supplement: Supplementary file 2 — Software build. Executable binary file of the software tool. (ZIP 118,784 kb) [file 12859_2018_2113_MOESM2_ESM.zip › cozoid/platform/docs/testng-6.5.1-javadoc.zip!/org/testng/IAttributes.html]

IAttributes (TestNG)


---


|  |  |  |
| --- | --- | --- |
| **Overview** \| **Package** \| **Class** \| **Tree** \| **Deprecated** \| **Index** \| **Help** | |  |
| **PREV CLASS**   **NEXT CLASS** | **FRAMES**    **NO FRAMES**     **All Classes** |  |
| SUMMARY: NESTED \| FIELD \| CONSTR \| METHOD | DETAIL: FIELD \| CONSTR \| METHOD |  |


---


## org.testng Interface IAttributes

**All Superinterfaces:**: java.io.Serializable

**All Known Subinterfaces:**: ISuite, ITestContext, ITestResult

**All Known Implementing Classes:**: SuiteRunner, TestRunner

---

``` public interface IAttributes extends java.io.Serializable ```

A trait that is used by all interfaces that lets the user add or remove their
own attributes.

---

| **Method Summary** | |
| --- | --- |
| `java.lang.Object` | `getAttribute(java.lang.String name)` |
| `java.util.Set<java.lang.String>` | `getAttributeNames()` |
| `java.lang.Object` | `removeAttribute(java.lang.String name)`             Remove the attribute |
| `void` | `setAttribute(java.lang.String name, java.lang.Object value)`             Set a custom attribute. |

**Method Detail**

### getAttribute

```
java.lang.Object getAttribute(java.lang.String name)
```

:   **Parameters:**: `name` - The name of the attribute to return

---


### setAttribute

```
void setAttribute(java.lang.String name,
                  java.lang.Object value)
```

:   Set a custom attribute.

---


### getAttributeNames

```
java.util.Set<java.lang.String> getAttributeNames()
```

:   **Returns:**: all the attributes names.

---


### removeAttribute

```
java.lang.Object removeAttribute(java.lang.String name)
```

:   Remove the attribute

    :   **Returns:**: the attribute value if found, null otherwise


---


|  |  |  |
| --- | --- | --- |
| **Overview** \| **Package** \| **Class** \| **Tree** \| **Deprecated** \| **Index** \| **Help** | |  |
| **PREV CLASS**   **NEXT CLASS** | **FRAMES**    **NO FRAMES**     **All Classes** |  |
| SUMMARY: NESTED \| FIELD \| CONSTR \| METHOD | DETAIL: FIELD \| CONSTR \| METHOD |  |


---
